# Comments on: Cardoso, C.F.; Drummond, A.F.; Lages, E.M.B.; Pretti, H.; Ferreira, E.F.; Abreu, M.H.N.G. The Dental Aesthetic Index and Dental Health Component of the Index of Orthodontic Treatment Need as Tools in Epidemiological Studies. *Int. J. Environ. Res. Public Health* 2011, *8*, 3277–3286

**DOI:** 10.3390/ijerph9093280

**Published:** 2012-09-07

**Authors:** Varun Arora, Narendra Kumar Gupta, Dilip K. Nath, Amrit Tandan, Pratik Chandra

**Affiliations:** 1 Active Research Group, APS, Lucknow 226003, India; 2 Department of Prosthodontics, Babu Banarasidas College of Dentistry, BBD University, Lucknow 227105, India; Email: drnkg19@gmail.com (N.K.G.); tandanamrit@yahoo.com (A.T.); 3 Department of Prosthodontics, Chandra Dental College and Hospital, Safedabad, Barabanki 225003, India; Email: dilip_nath2006@yahoo.co.in; 4 Department of Orthodontics, Saraswati Dental College and Hospital, Lucknow 227105, India; Email: pratikcagarwal@gmail.com

In regard to the article entitled “The Dental Aesthetic Index and Dental Health Component of the Index of Orthodontic Treatment Need as Tools in Epidemiological Studies” by Cardoso *et al.* [1] which checks the validity and agreement of two scales for orthodontic treatment need, we would like to draw your attention to a number of discrepancies in the design and methodology which have affected the results:

1. The authors have chosen a sample where the prevalence of orthodontic treatment need (according to the gold standard) is 91%. Unfortunately, this is not a true representation of the orthodontic treatment need of the population. In a literature review of studies published between 1951 and 2000 (in total 25 studies), covering orthodontic treatment needs in different ethnic groups, Thilander *et al.* [2] reported only 10 (40%) studies reporting orthodontic treatment need above 75% while only one (4%) study (Law *et al.*, [3]) in a Chinese population reported the prevalence of orthodontic treatment need above 90%, as reported by Cardoso *et al.* It is unfortunate that Cardoso *et al.* have chosen dental casts from the archive of Specialization Course in Orthodontics of a dental school—which indicates that these casts were prepared for the subjects who had undergone orthodontic treatment. This gives rise to one question *i.e.* whether the 9% of casts shown to be not requiring orthodontic treatment were *actually* of subjects who did not require orthodontic treatment? In our opinion, for such important research in which two scales were being evaluated and the study was carried out with the help of dental casts (*post-hoc* design), the valid option would have been to include a higher number of otherwise normal casts (as per the gold standard), as this was not a prospective study and could have been easily controlled. Otherwise, the study should have been carried out as a random evaluation in a sample drawn from the population.
2. The authors have shown the diagnostic accuracy of the two scales to be 61% and 67%, respectively. Now here we would like to provide a random simulation wherein the diagnostic accuracy will lead to an even higher result without using any scale in such a high prevalence situation. Suppose, using random criteria we propose all the 100% subjects to be requiring the treatment (Table 1).
  **Table 1:** Diagnostic Efficacy in high prevalence cases.

Random criteria	Gold standard		Total
	Yes	No	
Yes	91	9	100
No	0	0	0
Total	91	9	100
  Using these random criteria (which in fact are *no criteria*), owing to high prevalence the criteria has a diagnostic accuracy of 91%, sensitivity of 100% and specificity of 0%. This simulation shows that in case of a high prevalence, the accuracy of a model can be a wrong inference. The poor specificity as observed by Cardoso *et al.* in this article indicates that despite the use of proposed scales, the entire population has to be screened again by panel approach (gold standard) which shows that the time spent on administration of scales is of no use at all, rather it increases the overall time for treatment need assessment without adding any utility. Secondly, in such a situation, DHC cut-off 2 seems to be a better choice as at least it does have a 100% sensitivity, which means that the panel has not to go beyond the screened subjects.
3. In regard to reproducibility, we are amazed to see that the authors have used test-retest validation for only 10% of the samples, which merely turns out to be 13 models. It is difficult to assume that with the primary aim of the study being stated as “to assess the validity and reproducibility of the DAI and the DHC-IOTN in the identification of orthodontic treatment needs”, the reproducibility has been checked in only 13 observations.
4. Area under curve as depicted in Table 5 of the article (61% for DAI and 67% for DHC) indicates poor discrimination ability of the tests. In an ROC curve, an area of 1 represents a perfect test; an area of 0.5 represents a worthless test. According to rough estimates, an AUC value 0.60–0.70 shows poor accuracy of a diagnostic test, as no realistic classifier should have an AUC less than 0.5 (Fawcett [4]).

Under the circumstances, we do not agree with the conclusions of the authors that DHC and the DAI are reproducible and have reasonable accuracy and would recommend a rethinking of the applicability of DHC and DAI as per the results shown by the authors.
